# Utilizing AI to unveil the nonlinear interplay of convection, drift, and diffusion on galactic cosmic ray modulation in the inner heliosphere

**DOI:** 10.1038/s41598-022-25277-0

**Published:** 2022-12-01

**Authors:** Fadil Inceoglu, Alessandra Abe Pacini, Paul T. M. Loto’aniu

**Affiliations:** 1 Cooperative Institute for Research in Environmental Sciences, University of Colorado Boulder, Boulder, CO 80305 USA; 2 National Centers for Environmental Information, National Oceanographic and Atmospheric Administration, Boulder, CO 80305 USA

**Keywords:** Space physics, Magnetospheric physics, Solar physics

## Abstract

Galactic Cosmic Rays (GCRs) are charged particles, originating from galactic and/or extra-galactic Supernova Remnants (SNR), that continuously permeate the Heliosphere. The GCRs are modulated in the heliosphere by convection by solar wind (SW), drift via gradients and curvatures in the Heliospheric Magnetic Field (HMF), diffusion from fluctuations in the HMF, and adiabatic cooling in the expanding SW. An improved understanding of their modulation is imperative as studies on the variations in solar activity levels and solar eruptions in the past rely heavily on the relationship between their modulation and formation of the secondary particles in the Earth’s atmosphere. Here, for the first time, we utilize an AI method, Light Gradient Boosting Machines (LightGBM), to investigate the nonlinear interplay among the modulation processes in different timescales. Our study indicates that the nonlinear interplay among the mechanisms responsible for the GCR modulation in the inner heliosphere are not limited to the scenario of “drift-dominated solar minimum” versus “diffusion-dominated solar maximum”, instead they have dynamic behavior displaying variations in time and in timescales. This study also demonstrates the value of using AI methods to investigate non-linear physical processes in Space Physics in the era of big data.

## Introduction

The Galactic Cosmic Rays (GCRs) are charged particles with energies ranging from a few MeV to $10^{21}$ eV, with the maximum flux rates observed at energies around a hundreds of MeV^1^. They originate from galactic and/or extra-galactic Supernova Remnants (SNR) and they are thought to be initially accelerated via SNR shocks^2,3^. The GCRs primarily consist of fully ionized energetic atoms and sub-atomic particles that continuously permeates the heliosphere. The ionized atoms make 99% of the GCR flux, including Hydrogen ($\sim$ 87.1%), Helium ($\sim$11.9%), and nuclei of medium-heavy elements, such as Carbon, Nitrogen, Oxygen, and heavier Iron group elements (1%). The remaining 1% of the GCR flux is made of electrons, positrons, and antiprotons^4^. Upon entering the heliosphere, the transport of GCR particles with energies below about 30 GeV is modulated by the heliospheric magnetic field (HMF) and solar wind (SW) conditions, in a process called heliospheric modulation^5^. According to the basic transport equation^5,6^ (Eq. 1), modulation of GCR particles in the heliosphere (part a of Eq. 1) is a combination of convection of particles caused by the out-blowing SW (part b), drift of the cosmic ray particles in the heliospheric magnetic field caused by gradients and curvatures in the global HMF (part c), diffusion of particles due to fluctuations in the HMF (part d) and adiabatic cooling in the expanding SW (part e), given as:

1 $$\begin{aligned} \underbrace{\frac{\partial {f}}{\partial t}}_{a}= -(\underbrace{\mathbf{V}}_{b} + \underbrace{\langle \mathbf{v}_d \rangle }_{c}) \cdot \nabla {f} + \underbrace{\nabla \cdot ( \mathbf{K}_s \cdot \nabla {f} )}_{d} + \underbrace{\frac{1}{3}(\nabla \cdot \mathbf{V})\frac{\partial {f}}{\partial {ln}{P}}}_{e} \end{aligned}$$

where *f(r, P, t)* represents the cosmic ray distribution function, *P, t* and *r* denote rigidity, time and the position in 3 dimensions, respectively. The position in the 3-dimensional space is specified in a heliocentric spherical coordinate system with *r*, $\theta$ and $\phi$, where the equatorial plane is at a polar angle of $\theta$=90$^{\circ }$. Additionally, in the equation, **V** and $\langle \mathbf{v}_d \rangle$ are solar wind velocity and averaged particle drift velocity, while **K**$_s$ is the symmetrical diffusion tensor^5^.

The HMF influences the modulation of the GCRs through drift and the diffusion processes. An increase in the HMF strength results in a decrease in the drift velocities of the GCRs^7^. On the other hand, the turbulence in the HMF regulates the GCR diffusion in the heliosphere given by the diffusion coefficient (**K**$_s$) in Eq. (1), where the effective radial and perpendicular (in the polar direction) diffusion coefficients are given by $K_{rr} = K_{||}\,cos^{2}\Psi + K_{\perp r}\,sin^{2}\Psi$ and $K_{\theta \theta } = K_{\perp \theta }$, respectively^5^. In these equations, $K_{||}$ denotes parallel diffusion coefficient, while $K_{\perp r}$ and $K_{\perp \theta }$ show a perpendicular diffusion coefficient in the radial and polar directions, respectively^5^. The parallel and perpendicular diffusion coefficients are related to the corresponding mean free path (MFP, $\lambda$) length scales by $\lambda _{||,\perp } = 3K_{||,\perp }/v$, where *v* is the particle velocity^8^. The parallel and the perpendicular MFPs can be approximated via the relationships $\lambda _{||} \propto P^{2} / \delta B_{N}^{2}$ and $\lambda _{\perp } \propto (\delta B_{N}^{2})^{1/3} P^{2/3} / B_{0}^{4/3}$^9,10^, when neutron monitor (NM) data with rigidities $P \gtrsim$1 GV are used to study the modulation of the GCRs in the heliosphere^10^. In these relationships $B_0$ and $\delta B_N$ represent mean HMF magnitude and variance in the north-south (NS) component of the HMF, and *P* is the rigidity. Previous studies have shown that because of the solar cycle dependence of the HMF turbulence quantities, such as magnetic variances and correlation lengths, they reduce the drift effects on the GCR modulations^11^, which leads to the current theoretical understanding of drift vs diffusion dominated behavior of the GCR modulation in the inner heliosphere^12^. The HMF is created by the solar activity, more precisely dragging of the coronal magnetic field out into the heliosphere by the solar wind. The dynamic conditions in the solar corona are controlled by the coronal magnetic field governed by the photospheric magnetic fields and plasma motions^13^. The synoptic observations of the solar photospheric magnetic field can be used to infer the coronal magnetic field by calculating the open solar magnetic field strengths using a method called Potential Field Source Surface (PFSS)^14^. This method extrapolates the photospheric magnetic field to scales greater than 2.5$R_{\odot }$, where the magnetic field lines are assumed to be open and radial.

Due to the heliospheric modulation, the GCR fluxes measured near Earth show quasi-periodic variations. The most prominent period that can be observed in the GCR intensities is the $\sim$ 11 year Schwabe cycles^15^, also known as the solar cycles (SC). The variations in the Schwabe timescales are closely related to the variations in the solar wind speed and the HMF magnitudes, which vary in-phase with the Schwabe cycles^16^. A physical explanation for the GCR modulation in the Schwabe timescales comes from propagating barriers formed by the solar wind and magnetic field co-rotating structures, which in turn obstruct the GCRs propagation into the inner heliosphere through outward-propagating diffusive barriers^5,17^. Further, another interesting periodicity observed in the GCR flux is the $\sim$22 year cycle, which is directly connected to the Hale polarity cycle^18^. These variations are attributed to the drift of the GCRs based on the polarity of the large-scale solar magnetic field^7^. When the heliospheric polarity is positive, the positively charged GCR particles drift inward from the heliospheric poles and outward along the Heliospheric Current Sheet (HCS), while they experience an easier inward drift along the HCS and outward along the heliospheric poles when the heliospheric polarity is negative^5,7^. The HCS is a large-scale magnetic boundary that separates the opposite polarities of the large-scale dipolar magnetic field of the Sun, expanding throughout the heliosphere^19^.

The role of drift as the dominant solar modulation in the inner heliosphere (<10 astronomical units) during solar minima and the diffusion during solar maxima became an accepted conceptual paradigm based on ground- and space-based observations for the last solar cycles. However, this scenario changed dramatically around the onset of the solar cycle (SC) 24^20^. Additionally, using neutron counting rates (NCRs), as indicators of the GCR flux at Earth, it was previously shown that the GCRs experienced less efficient heliospheric modulation during both maximum and minimum of the SC-24, suggesting a recent decrease of the HMF turbulence^21^, which agrees with results drawn from models and space-based data^22–26^. These results point to a recently established new balance among GCR modulation processes.

Even though the main drivers and their effects on the modulation of the GCRs in the inner heliosphere are considered well-known^27^, due to their nonlinear natures, the relative importances of the convection, drift, diffusion, and adiabatic cooling and detailed understanding of the effects of these drivers in different timescales are still an ongoing research^27–31^. Better understanding of the modulation of GCRs in the heliosphere is crucial as studies on the past variations in solar activity, including subdecadal and annual reconstructions of sunspot numbers as a solar activity proxy, as well as studies on the historic solar eruptions rely heavily on the relationship between the secondary particles that are created in the Earth’s upper atmosphere by the GCRs and their modulation^32–39^.

Therefore, answering the questions of how GCRs are modulated in the inner heliosphere in different timescales and how the relative importances of the modulation mechanisms, such as diffusion, convection, and drift, vary in time, is of great importance. In this study for the first time, we utilize a machine learning method, Light Gradient Boosting Machine (LightGBM) regressors^40^ and permutation importance (PI) scores^41^ to catch the nonlinear interactions and relative importances of these modulation mechanisms. For this purpose, we use data from various sources, extending from the solar surface to near Earth, as proxies for drift, diffusion, and convection mechanisms, in LightGBM regressors in an attempt to answer these questions.

## Results

### Identifying the heliospheric drivers of the GCR modulation: LightGBM and Permutation Importances

To investigate the relative importances of the modulation mechanisms of the GCRs, as indicated by NCRs, in the inner heliosphere, we use data in two different timescales; (1) unfiltered data that includes variations in all timescales and (2) time scales below 5 years that captures higher-frequency variations, which might be suppressed by longer-term variations. The data used in our study reflect variations in the heliospheric conditions causing the modulation of the GCRs in the inner heliosphere, *i. e.* equatorial ($|B_{eq. dip.}|$) and axial ($|B_{ax. dip.}|$) open solar magnetic fields that are sensitive to the low-latitude coronal holes and polar coronal holes, respectively, as proxies for the outward-propagating diffusive barriers. The HCS tilt angle ($|R_{av}|$) is used as a proxy for the GCR drift processes, while solar wind speed at Lagrangian-1 point (L1) ($|v_{sw}|$) is a proxy for the convection of the GCRs near Earth. The HMF turbulence at L1 ($\delta B_{N}^{2}$) and parallel ($\lambda _{||}$) and perpendicular ($\lambda _{\perp }$) MFPs were adopted as a proxy for diffusion of the GCRs. The magnitude of the average HMF vector ($|B_{av}|$) at L1 influences both drifts and diffusion of the GCRs in the inner heliosphere. We also included the solar wind dynamic pressure as it affects the radial position of the heliopause^42^, which in turn influences the GCR modulation^43^.

**Figure 1:**
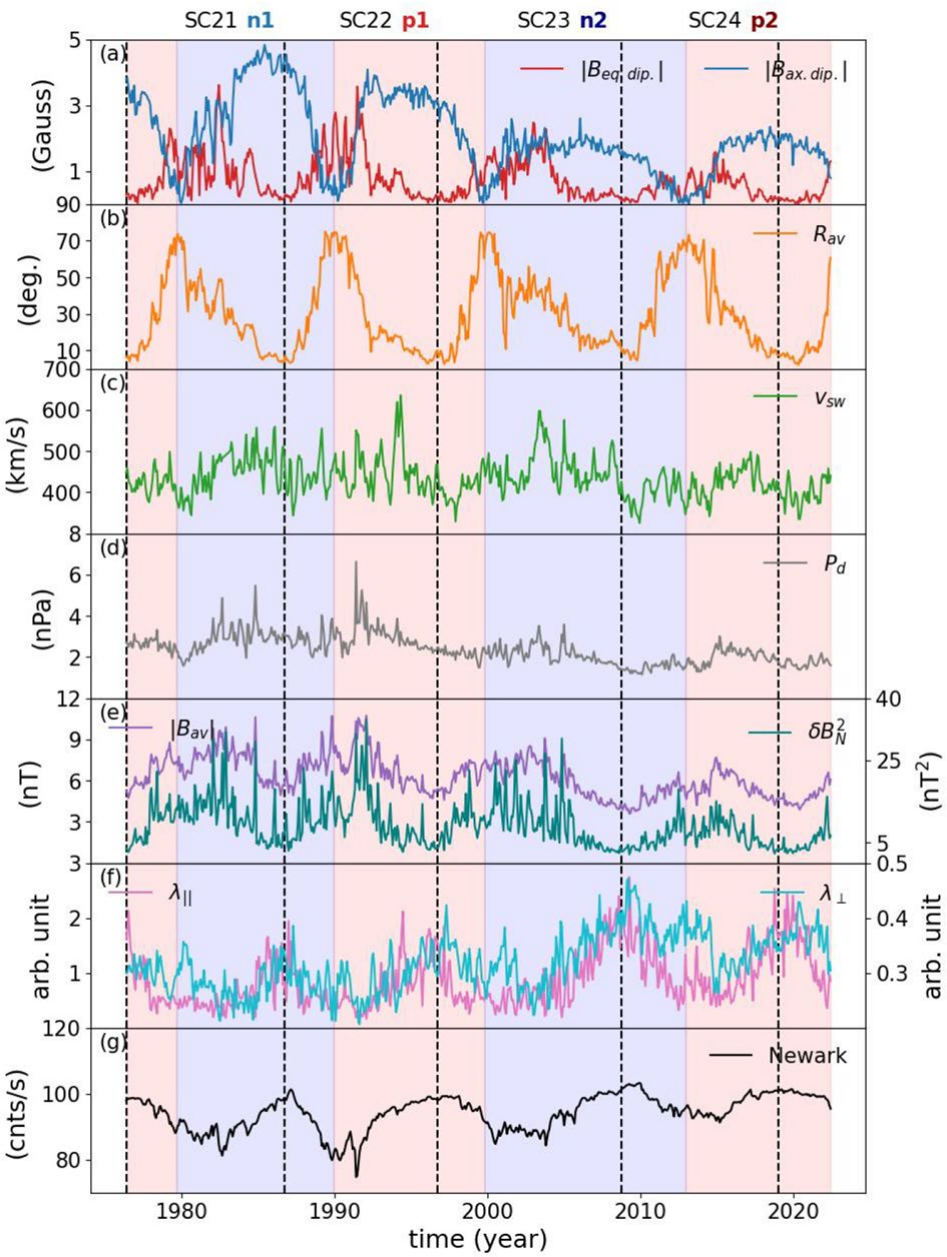
Panel (**a**) displays the magnitudes of the axial (blue) and equatorial (red) dipolar magnetic fields. Panel (**b**) shows the average tilt angle of the HCS and the average solar wind speed is shown in panel (**c**). Panel (**d**) shows the solar wind dynamic wind pressure, while panel (**e**) shows the field magnitude average (purple), and the variance in the north-south component in the HMF (teal). Panel (**f**) shows the parallel (pink) and perpendicular (cyan) MFPs of the particles. Panel (**g**) shows the Newark NCRs. The blue and red shaded areas show when the HMF has negative and positive polarities, respectively. The vertical dashed lines indicate the solar minimum dates. We also note the solar cycles and the positive and negative polarity intervals.

The magnitude of the axial dipolar field of the Sun, $|B_{ax. dip.}|$, which is sensitive to the polar coronal holes, shows an anti-phase relationship with the Schwabe cycles attaining its maximum values during solar cycle minimum conditions. The magnitude of the equatorial dipolar field ($|B_{eq. dip.}|$), on the other hand, which is sensitive to low-latitude coronal holes, displays an in-phase relationship with the Schwabe cycles attaining its maximum values during the solar cycle maximum (Fig. 1a). Similar to the magnitude of the equatorial dipolar field, the HCS tilt angle, $R_{av}$, displays variations in-phase with the SCs (Fig. 1b). The HCS tilt angle is defined as the average of the maximum northern and southern latitudinal excursion of the HCS at a given distance from the Sun^44^. The SW speed, $v_{sw}$, displays an abrupt decrease on the onset of SC-24 preceded by a stable long-term trend with higher-frequency fluctuations superimposed (Fig. 1c). The solar wind dynamic pressure ($P_d$) displays in-phase relationship with the equatorial dipolar field with a long-term decreasing trend in its amplitude over the last four SCs (Fig. 1d). The magnitude of the average field vector ($|B_{av}|$) and the turbulence ($\delta B_{N}^{2}$) display an in-phase relationship with the SCs (Fig. 1e). The parallel and perpendicular MFPs of the particles show an anti-phase behavior with the SCs. Instead, they display an in-phase relationship with the axial dipolar field and the amplitudes of the parallel MFPs show anti-correlation with those of the axial dipolar field (Fig. 1f). The NCRs, as expected, show an inverse correlation with the SCs, attaining their maximum during solar minimum conditions (Fig. 1f). The effects of the solar magnetic polarity in the propagation trajectory of the GCRs through the heliosphere can be observed as flat-topped and peaked-topped maxima in the NCRs (Fig. 1g).

Importantly, while axial and equatorial dipolar open magnetic field of the Sun together with the magnitude of the average HMF vector, the turbulence, and the solar wind dynamic pressure show a long-term decrease trend in their strength over the course of the past 4 SCs, the HCS tilt angle and solar wind speed are observed to be more stable in magnitudes, while the amplitude of the parallel and perpendicular MFPs have continuously increased. Similarly, the GCR intensities, represented here by the NCRs, show an increasing trend (Fig. 1).

**Figure 2:**
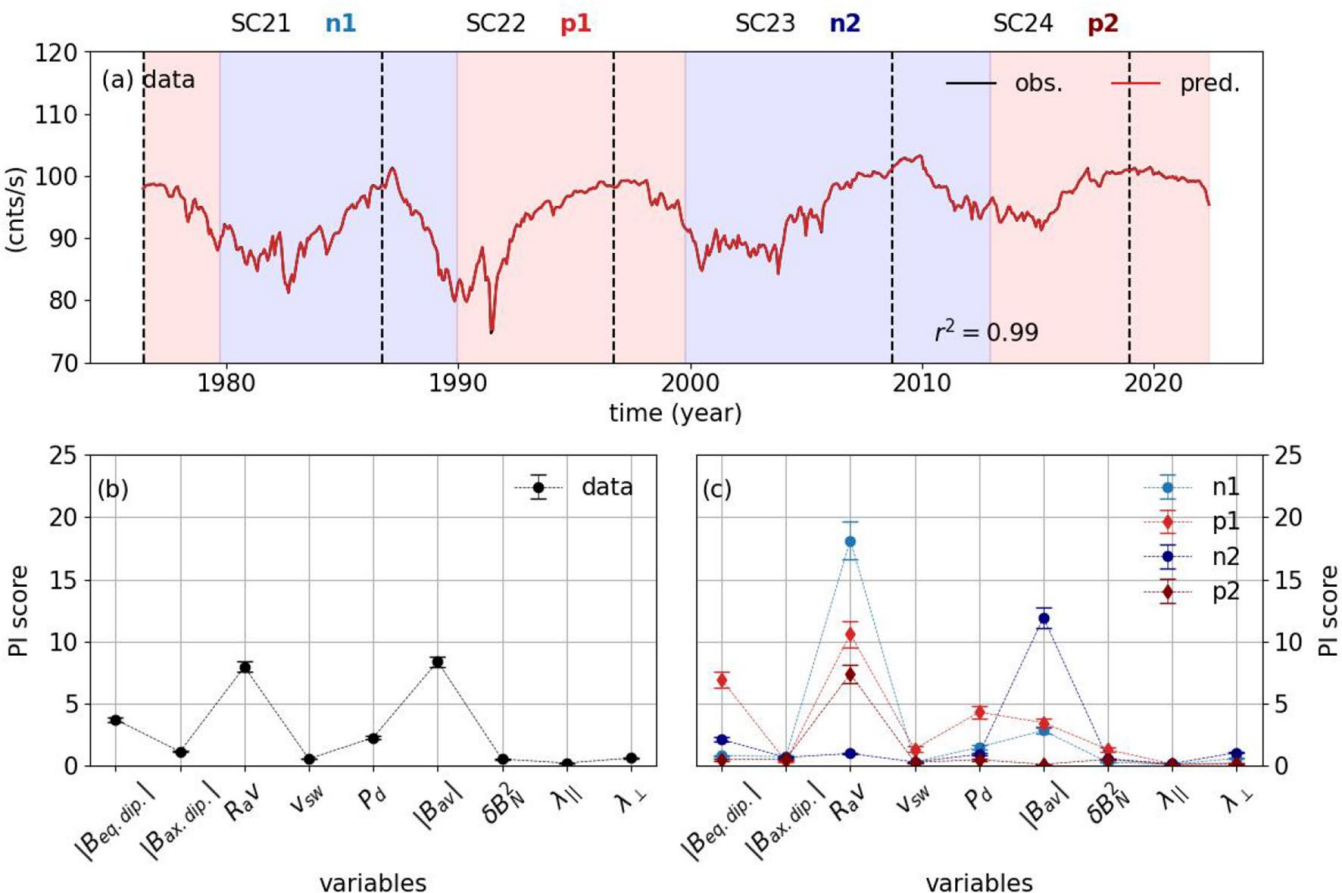
Panel (**a**) shows the LightGBM fit (red) plotted on the Newark NCRs (black). Permutation Importance scores for the unfiltered data for the past 4 solar cycles (**b**), and for the positive and negative polarity cycles (**c**). The blue and red shaded areas show when the HMF has negative and positive polarities, respectively. The vertical dashed lines indicate the solar minimum dates. We also note the solar cycles and the positive and negative polarity intervals.

Using the heliospheric drivers, as proxies to the modulation mechanisms of the GCRs in the inner heliosphere, in the LightGBM regressors, we could explain the 99% of the variability in the unfiltered NCRs (Fig. 2a). The LightGBMs were successful at capturing the peaked- and flat-topped maxima seen in the NCRs, which are related to the solar magnetic polarity as well as to reproduce the higher frequency variations especially (Fig. 2a). To investigate the relative importances of each heliospheric driver, we use their Permutation Importance (PI) scores. The most important factor in reproducing the observed NCRs is observed to be the magnitude of the average field vector ($|B_{av}|$), which is closely followed by the HCS tilt ($R_{av}$), and equatorial dipolar field of the large-scale open solar magnetic field ($|B_{eq. dip.}|$) and solar wind dynamic pressure (Fig. 2b). Another important feature observed in the PI scores is that the axial dipole ($|B_{ax. dip.}|$), solar wind speed ($v_{sw}|$), parallel ($\lambda _{||}$) and perpendicular ($\lambda _{\perp }$) MFPs, and the turbulence ($\delta B_{N}^2$) have very small contributions in reproducing the NCR variability.

Additionally, we investigated the relative importances of the solar and heliospheric data as proxies to the GCR modulation mechanisms in each solar polarity intervals between the solar cycles, i.e. n1, p1, n2, and p2 denote the negative polarity between $\sim$ 1980 and $\sim$ 1990, the positive polarity between $\sim$ 1990 and $\sim$2000, the negative polarity between $\sim$ 2000 and $\sim$ 2014, and the positive polarity since then, respectively. The results show that the HCS tilt ($R_{av}$) is the main contributor for n1, p1, and p2 intervals, which correspond to the periods between SC-21 and 22, SC-22 and 23, and SC-24 and 25. During p1, different from the other intervals, the importance of the equatorial dipole open solar magnetic field ($|B_{eq. dip.}|$) is the second highest, which is followed by the solar wind dynamic pressure ($P_d$) and the magnitude of the average field vector ($|B_{av}|$), and the turbulence ($\delta B_{N}^2$). However, for the n2 interval, which is between SC-23 and 24, the average field vector ($|B_{av}|$) takes over the HCS tilt becoming the most important factor (Fig. 2c), which is followed by the equatorial dipole open solar magnetic field ($|B_{eq. dip.}|$), the HCS tilt ($R_{av}$), and the perpendicular MFPs ($\lambda _{\perp }$)). However the importances of the latter three are much smaller compared with the the magnitude of the average field vector ($|B_{av}|$). Overall, one can argue that the HCS tilt angle is the most dominant factor when the polarity intervals are individually investigated ad its importance was slightly reduced between SC-22 and 23, and its importance became the forth highest during n2 between SC-23 and 24, after which its importance is restored as the most important factor (Fig. 2c).

**Figure 3:**
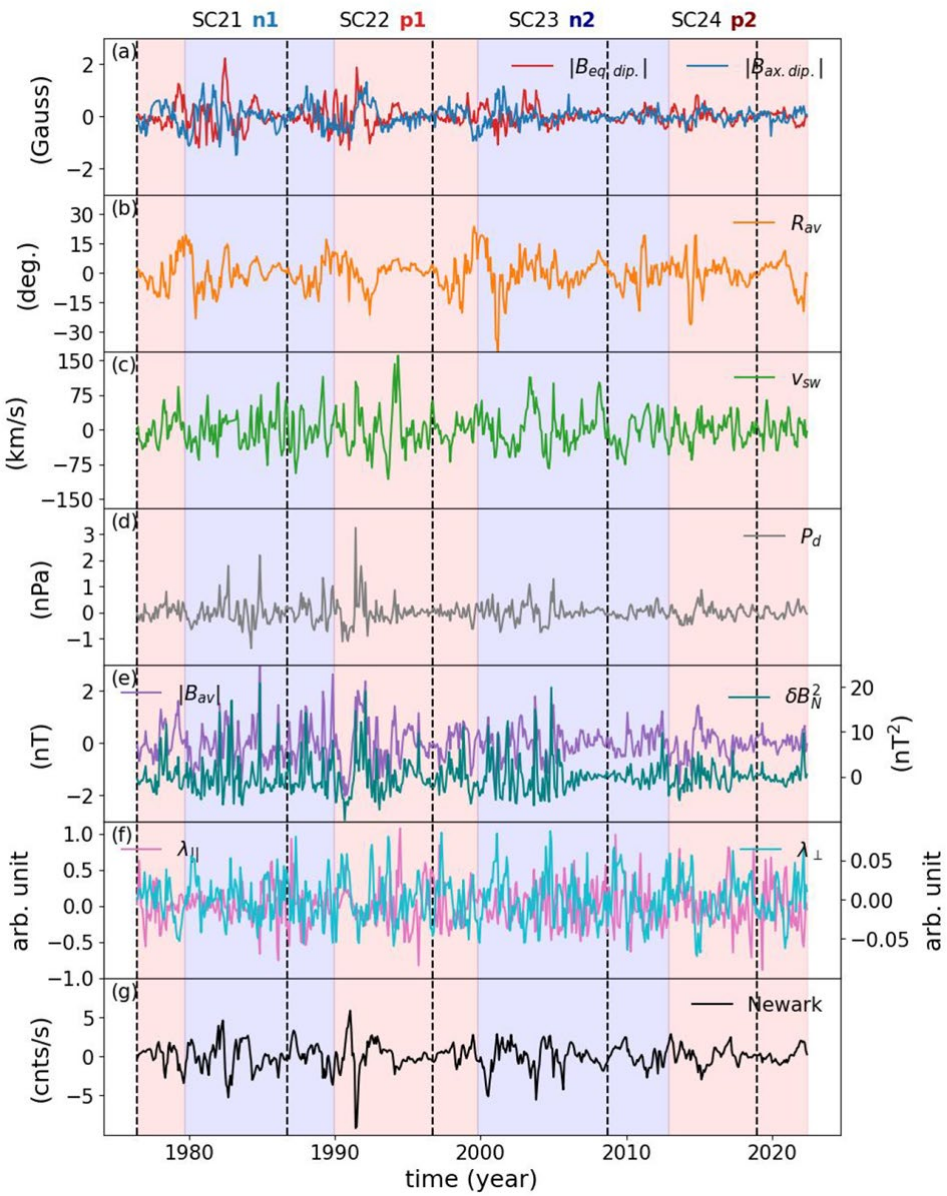
Same as Fig. 1 but high-pass filtered using a cut-off frequency of 5 year$^{-1}$.

To investigate how the higher-frequency modulation of the NCRs responding to the heliospheric drivers, we performed the same analyses to the data that are high-pass filtered with a cutoff frequency of 5 year$^{-1}$ (Fig. 3).

The high-pass filtered data do not exhibit any variations in or out of phase with the Schwabe or Hale cycles (Fig. 3). The amplitude of variations in the equatorial and axial dipolar open solar magnetic field is higher in SC-21 and 22, while it becomes smaller through SC-24 (Fig. 3a). The HCS tilt angle in this time scale shows a sharp decrease around 2000 after the polarity changes from positive to negative, which can also be observed around 1980 with a shallower dip (Fig. 3b). The most interesting feature is the sudden decrease in the NCRs around 1991, coinciding with a large CME^45^, which coincides with the sudden increase in the equatorial dipolar open magnetic field of the Sun that is also aligned with the sudden jumps in the solar wind speed, solar wind dynamic pressure, the average field vector magnetic field, and turbulence (Fig. 3a, c, d, e, and g). On the contrary, the dip observed around 1982, which overlaps with the 1982 solar storms^46,47^, although is aligned with the sudden increase in the equatorial dipolar open magnetic field of the Sun, there is no sudden jumps observed in the solar wind speed or the average field vector magnetic field. This dip, however, aligns with the sudden jump in the HCS tilt angle (Fig. 3a, b, and f). There are also other sudden dips in the NCRs in this timescale, which are, similar to those observed in 1982 and 1991, related to severe solar storms. Another interesting feature is the sudden jump in the average field vector magnetic field in late 1984, which is followed by a sudden increase in the parallel MFPs of the GCRs in mid 1985 with a lag of a few months (Fig. 3e and f).

**Figure 4:**
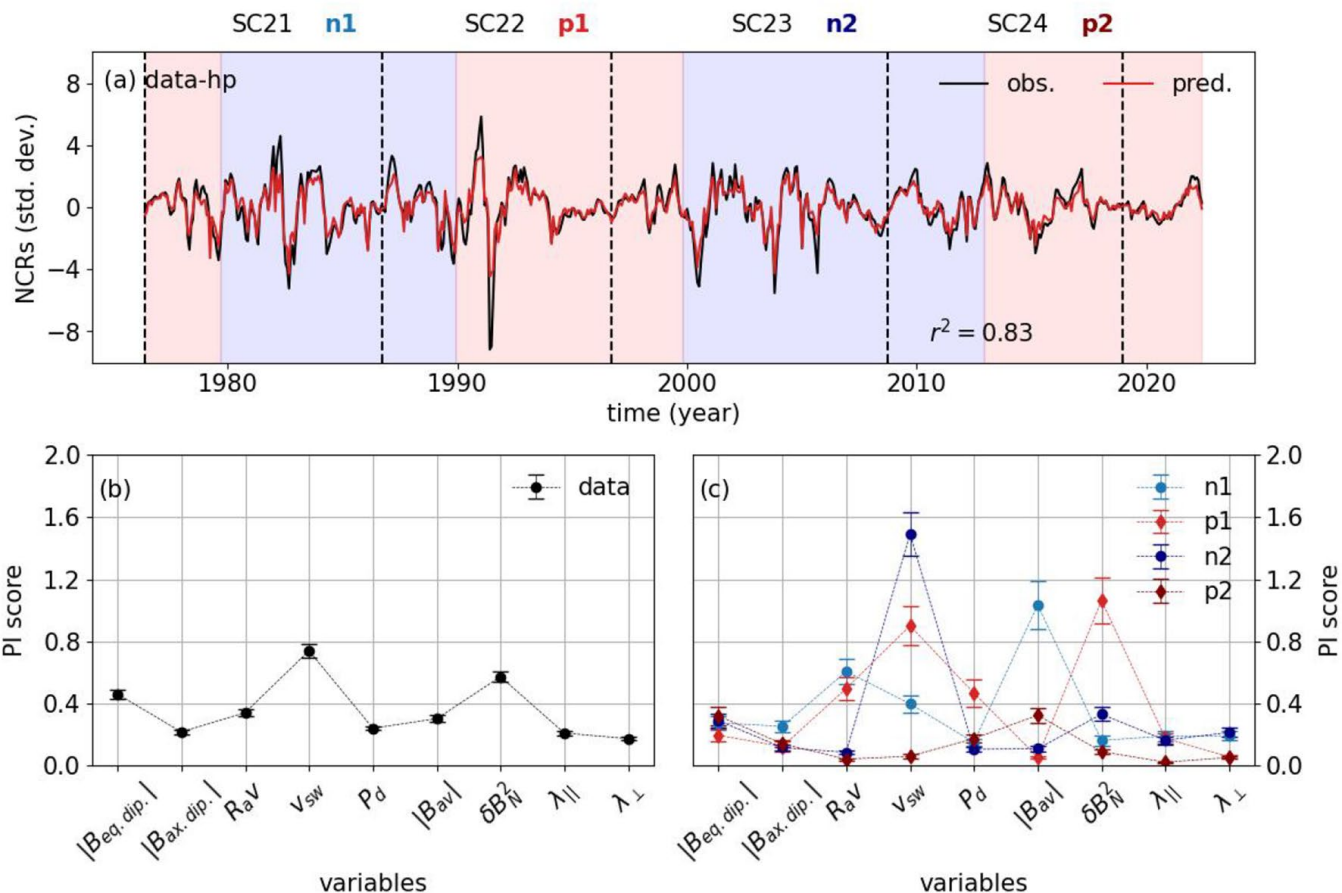
Same as Fig. 2, but for the high-pass filtered data.

Similar to the unfiltered data, we also used a LightGBM regressor to reconstruct the NCRs in this timescale and studied the relative importances of each factor using their PI scores during the full duration of the study as well as during each polarity interval (Fig. 4). Overall, the 83% of the variations in the NCRs can be explained by the solar and heliospheric data we use in this study (Fig. 4a). For the full duration covering the period from 1978 to 2022, the most effective factor is the solar wind speed ($v_{sw}$) followed by the turbulence ($\delta B_{N}^2$), and the equatorial dipolar open solar magnetic field ($|B_{eq. dip.}|$). Different from what is observed in the unfiltered data, in this timescale the parallel and perpendicular MFPs of particles have more contribution in reconstructing the variations in the NCRs, however their contributions are still smaller than those from the HCS tilt, the average field vector magnetic field, solar wind dynamic pressure, and axial dipolar open solar magnetic field ($|B_{ax. dip.}|$) (Fig. 4b). The picture becomes a little different when we consider the polarity intervals individually. The contributions from each factor can clearly be observed for each of the polarity intervals under consideration. For the n1, which is between SC-21 and 22, the contribution from the average field vector magnetic field ($|B_{av}|$) surpasses the other factors, and it is followed by the HCS tilt ($R_{av}$) and the solar wind ($v_{sw}$) (Fig. 4c). This situation, however, changes for p1, where the turbulence ($\delta B_{N}^2$) becomes the most dominant contributor, closely followed by the solar wind speed ($v_{sw}$), the HCS tilt ($R_{av}$), and solar wind dynamic pressure ($P_d$). During this period, the rest of the factors contribute to the NCR variations almost equally. For the n2, although the solar wind ($v_{sw}$) is the major factor, the contribution from other factors, such as the HCS tilt angle ($R_{av}$) and axial dipolar open solar magnetic field ($|B_{ax. dip.}|$), become weaker. During this period, onthe other hand, the contributions from the turbulence, the equatorial dipolar open solar magnetic field and parallel and perpendicular MFPs increase. As for the p2, the main contributor is the equatorial dipolar open solar magnetic field ($|B_{eq. dip.}|$) and the average field vector magnetic field ($|B_{av}|$), while the contribution from the solar wind speed ($v_{sw}$) and HCS tilt angle ($R_{av}$) are minimum (Fig. 4c).

## Discussion

One of the most striking features observed for the modulation of the GCRs in the unfiltered data is the interplay between the drift and diffusion mechanisms, as indicated by the equatorial dipolar open solar magnetic field and average field vector magnetic field, and the HCS tilt angle. During the negative polarity interval between $\sim$1980 and $\sim$1990 (n1), the GCRs are modulated mainly by the drift process, as indicated by the HCS tilt angle, which is followed by the diffusion, as indicated by the average field vector magnetic field, which has important effects on both the drift and the diffusion processes^30^. For the following positive polarity interval between $\sim$1990 and $\sim$2000 (p1), however, the role of the drift process becomes slightly weaker, while the effects of the diffusion process becomes stronger, as indicated by the increased importances of the equatorial dipolar open solar magnetic field, solar wind dynamic pressure, and the average field vector magnetic field, that are sensitive to the existence of the low-latitude coronal holes that create the outward-propagating diffusive barriers modulating the GCR flux in the inner heliosphere^13,17^. The physical explanation for this comes from the fact that the equatorial dipolar open solar magnetic field is linked to the emerging active regions and their decay products, such as coronal holes^17^. During solar cycle maximum conditions the topology of the HCS is very complex^48^ and the solar wind speed shows longitudinal variations^13^. In combination with the rotation of the solar wind source regions, these conditions lead to compression of the fast winds onto slow winds ahead, forming the stream interaction regions, which corotate with the Sun in the quasi-steady regime, the so-called corotating interaction regions (CIRs)^49^. These CIRs will obstruct the GCR propagation into the inner heliosphere via diffusion processes^5,17^. For the negative polarity interval between $\sim$ 2000 and $\sim$ 2014 (n2), the interplay between the drift and diffusion processes in modulating the GCR flux in the inner heliosphere is indistinguishable based on the PI scores of the average field vector magnetic field, the equatorial dipolar open solar magnetic field, and the HCS tilt angle, respectively. Even though the diffusion and drift processes can be approximated by the equatorial dipolar open solar magnetic field and the HCS tilt angle, their PI scores are very low in comparison to the highest PI score that is calculated for the average field vector magnetic field, which has an effect in both the drift and the diffusion processes^30^. For the following positive polarity interval between SC-24 and 25, after $\sim$2014 (p2), which is still ongoing, the drift is the major mechanism, as indicated by the highest PI score calculated for the HCS tilt angle, in modulating the GCR flux, whereas there only minimal contribution from the other factors. This might be related to the weakened solar activity that started in SC-23. However, it must be noted that SC-24 is still ongoing and these results might change after the polarity reversal.

Another striking feature is the suppression of the convection of the GCRs in the inner heliosphere by the drift and diffusion processes in timescales longer than 5 years. The solar wind speed is known to affect both the convection of the GCRs outward in the heliosphere and the adiabatic energy loss rates of the GCRs (see part-e in Eq. 1), which becomes quite large in the inner heliosphere^5^. The greater solar wind speed leads to increased outward convection and increase the adiabatic cooling energy loss rate^30^. For timescales shorter than 5 years, the contribution of the convection and adiabatic cooling energy loss processes in GCR modulation becomes more visible. For the negative polarity period between $\sim$ 1980 and $\sim$ 1990 (n1), the drift and diffusion mechanisms are still major contributors to the GCR modulation, while the convection has a secondary effect. However, for the following two polarity cycles between $\sim$ 1990 and $\sim$ 2014 (p1 and n2), the convection and adiabatic cooling energy loss via solar wind take over the GCR modulation, while drift and diffusion processes work in the background. Interestingly, for the positive polarity period between SC-24 and 25 (p2), which is still ongoing, the convection process in modulation of the GCRs in the inner heliosphere becomes the weakest, while diffusion via out-ward propagating diffusive barriers, together with drifts, plays a bigger role. This could be explained by the abrupt decrease observed for the solar wind speed after $\sim$ 2010 on the onset of SC-24 (Figs. 1c and 3c), while the amplitude of variation in the solar wind speed before $\sim$ 2010 is higher, leading to stronger modulation of GCRs in the inner heliosphere by convection and adiabatic energy loss.

Using a more traditional method, such as multi-variable regressions that is not sensitive to nonlinear interactions, it was shown that the drift mechanism during SC-24 is lower, while the effects of diffusion continuously grow through SC-23 and-24^50^. Our results, however, are in contradiction with these previous findings. Our results drawn from the LightGBMs and PIs, which are better suitable for nonlinear processes, suggest that with the declining solar activity, especially after the maximum of SC-23, the modulation effect of diffusion became less important, while drift effects became more prominent. The physical explanation for this comes from the lack of emergence of stronger active regions and their decay products, such as coronal holes, leading to faster solar winds and CIRs and outward propagating diffusive barriers, whereas the drift of GCRs along the HCS due to gradients and curvature in the HMF^8^. Our results on the drift effects on GCR modulations in long and short timescales are in-line with those drawn from Empirical Mode Decomposition, showing that the contribution of the drift effects are much higher in the 11-and 22-year timescales compared with the 6-year timescale^51^.

In this study, we used LightGBMs to investigate the nonlinear interplay among the modulation mechanisms of the GCRs in the inner heliosphere. Our results suggest that investigations on GCR modulation mechanisms must consider their relative importances in different timescales. We observed that the contributions of the outward convection and the adiabatic energy loss to the modulation of the GCRs in the inner heliosphere are suppressed by the drift and diffusion mechanisms for timescales longer than 5 years. This might be caused by global effects of the open solar magnetic field through outward propagating diffusive barriers and the very dynamic topology of the HCS. For periods shorter than 5 years, these variations are much slower and therefore have smaller magnitudes compared with the higher-frequency and more dynamic fluctuations observed in the solar wind speed. However, it must be noted that in this timescale, we were able to explain only the 83% of the variations in the GCR intensities for the full duration of the period and the remaining of which might be related to the occurrence rates and frequencies of the solar eruptions, such as CMEs and interplanetary shocks^46,52^.

In conclusion, our study indicates that the nonlinear interplay among the mechanisms responsible for the GCR modulation in the inner heliosphere are not limited to the scenario of “drift-dominated solar minimum” versus “diffusion-dominated solar maximum”, instead they have dynamic behavior displaying variations in time and in timescales. For periods longer than 5 years, the most prominent mechanisms that modulates the GCRs are observed to be the drift and the diffusion mechanisms, which are approximated via the HCS tilt angle and the equatorial dipolar open magnetic field of the Sun. For periods shorter than 5 years, this picture changes where the convection and adiabatic cooling via solar wind and diffusion via HMF plays a major role almost throughout the study period, while modulation via drift works more in the background. Additionally, this study demonstrates the value of using AI methods to investigate non-linear physical processes in Space Physics in the era of big data.

## Methods

### Extracting the dipolar open solar magnetic field data from Wilcox Solar Observatory

For variations in solar activity levels, we used synoptic photospheric magnetic field maps of the radial magnetic field for each Carrington Rotations (CRs) obtained from line-of-sight magnetograms from the WSO (http://wso.stanford.edu)^53,54^ for variations in solar activity levels. The synoptic map data extends from CR1642 (May 1976) to CR2258 (June 2022) covering the past 4 solar cycles (SCs). To further investigate the modulation effects of the different components of the large-scale solar magnetic activity, we decomposed the synoptic maps based on spherical harmonic analysis using the Legendre-transform software provided by the potential field source surface package (PFSS) of SolarSoft software package^55,56^. PFSS method assumes zero current density in the corona, and uses the radial synoptic photospheric magnetic maps as the inner boundary condition, while the outer boundary is the source surface, typically placed above 2.5R$_{\odot }$, where the magnetic field lines are assumed to be radial and open^14^. For each CRs, we then obtained complex coefficients $B_{\ell }^{m}(t)$ for spherical harmonic degrees $\ell = 0,..., 60$, where $\ell$, and *m* denote spherical harmonic degree and azimuthal order, respectively. These complex coefficients are proportional to the amplitudes of each spherical harmonic mode $Y_{\ell }^{m}$;

2 $$\begin{aligned} B_{r}(\Theta ,\phi ,t) = \sum _{\ell =0}^{\ell _{max}} \sum _{m=0}^{\ell } B_{l}^{m}(t) Y_{l}^{m}(\Theta ,\phi ) ,\end{aligned}$$

where $\Theta$, $\phi$, and *t* denote colatitude, longitude, and, time, respectively, on the source surface. Degradation of the higher spherical harmonic degrees are a known issue^57,58^, therefore we chose to limit the spherical harmonic degrees to $\ell =1$. Following these steps, we calculated the magnitudes of the equatorial dipolar ($\ell =1, |m|=1$) and axial dipolar ($\ell =1, m=0$) open solar magnetic fields. Additionally, we used the averaged HCS tilt angles from the WSO^59^ (http://wso.stanford.edu/Tilts.html) calculated using the radial boundary condition at the photosphere for each CR.

### NCRs as indicators of GCR variability

For variations in the GCR intensities, we used monthly averaged pressure-corrected NCRs from the Newark Neutron Monitor (NNM) (University of Delaware Department of Physics and Astronomy and the Bartol Research Institute, USA). The NNM is located at 39.68 N and 75.75 W and at an altitude of 50 m with an effective vertical cut-off rigidity of $P=$2.40 GV. All NCR data are obtained from the Neutron Monitor Data Base (NMDB, http://www01.nmdb.eu/). The Newark NM included one missing data point in March 1978. Similar to the OMNI2 data, we used a spline function of order 1 to fill the missing data. Then we mapped the monthly averaged NCRs from the NNM onto CR time resolution.

### Extracting SW and HMF information from OMNIWeb data

For variations in the HMF, we used extended OMNI2 data set, which provides hourly averaged solar wind speed, solar wind dynamic pressure, and the magnetic field magnitude average and its components (https://omniweb.gsfc.nasa.gov)^60^. These measurements are made near Earth, at the Lagrangean-1 point. To have the same time resolution with the solar magnetic field data from the WSO, we calculated the monthly averages of the solar wind speed and the HMF data, and then mapped the values for each CR spanning the time period from May 1976 to June 2022. There were 5 data points from around September 1982 to January 1983 missing in the solar wind and dynamic pressure data. We used a spline function of order 1 to interpolate to fill the missing data. Further, we calculated the parallel and the perpendicular MFPs of the particles with rigidities $P \gtrsim$1 GV using the relationships $\lambda _{||} \propto P^{2} / \delta B_{N}^{2}$ and $\lambda _{\perp } \propto (\delta B_{N}^{2})^{1/3} P^{2/3} / B_{0}^{4/3}$ where $B_0$ and $\delta B_N$ represent mean HMF magnitude and variance in the north-south (NS) component of the HMF, and *P* is the rigidity^9,10^. The variance in the NS component of the HMF is calculated using the hourly magnetic field data for monthly intervals^61^. Then we mapped the monthly values onto CR time resolution.

### Preprocessing data

To investigate the GCR modulation in different timescales we filtered the data using a Butterworth filter of order 5 with a cutoff frequency of 5 yr$^{-1}$ as the longer-term variations in the data could suppress the relationships in the high-frequency range.

### LightGBM regressors and permutation importances

To calculate the relative importances of convection, drift, and diffusion, we utilized LightGBM as regressors and calculated the importances using PIs. LightGBM is a high-performance ML algorithm based on decision trees, which allows high-efficiency, parallel and distributed learning^62^. LightGBM uses different types of boosters, such as Gradient Boosting Decision Tree^63^ (gbdt) and Dropouts meet Multiple Additive Regression Trees^64^ (dart). These boosters differ in advantages and disadvantages, such as accuracy, stability, converge times, memory usage. For example, let $X^s$ and *G* represent the input and the gradient spaces, respectively. The training set with a dimension *s* in space $X^s$ has *n* independent and identically distributed instances such as $\{x_1, x_2, x_3, \dots , x_n \}$. The gbdt approach uses decision trees to learn a function from the given $X^s$ and to the *G* by iterating the gradient boosting, where the negative gradients of the loss function in each iteration calculated according to the output of the model are denoted as $\{g_1, g_2, g_3, \ldots , g_n \}$. The decision tree then splits each node where it gains the largest information, which is measured by the variance after the splitting calculated as follows^62,63^:

3a $$\begin{aligned} V_{j|O}(d) = \frac{1}{n_O}\left( \frac{\left( \sum _{\{x_i\in O:x_{ij}\le d\}}g_i \right) ^{2}}{n_{l|O}^{j}(d)} + \frac{\left( \sum _{\{x_i\in O:x_{ij}> d\}}g_i \right) ^{2}}{n_{r|O}^{j}(d)} \right) \end{aligned}$$

where,

3b $$\begin{aligned} n_O&= \sum I[x_i\in O] \end{aligned}$$

3c $$\begin{aligned} n_{l|O}^{j}(d)&= \sum I[x_i\in O:x_{ij}\le d] \end{aligned}$$

3d $$\begin{aligned} n_{r|O}^{j}(d)&= \sum I[x_i\in O:x_{ij}> d] \end{aligned}$$

The decision tree, then, selects $d_j = argmax_dV_j(d)$ for feature *j* and calculates the largest gain $V_j(d_j^*)$, where the data will be split into the right and left child nodes, accordingly^62,63^. To optimize the LightGBM hyper-parameters, such as the booster type, learning rate, number of estimators, number of leaves, and so on, we used Bayesian optimization of *scikit-optimize*^65^ python library. The hyper-parameter optimization is done separately for each timescale; unfiltered and high-passed data, separately (Table 1). Bayesian optimization, different from manual or automated grid search algorithms that uses all possible permutations of the hyper-parameter settings, use predetermined number of parameter settings is drawn from each specified distributions for the hyper-parameters. In our study, we separate the data into training (75%) and test (25%) subsets. For the training set, we use 50 iterations for each parameter setting, 10-fold cross validation for better optimization of the hyper-parameters, and negative mean squared error for scoring. We then tested the LGBM fits on the test data as well as the whole period covering the time between May 1976 and June 2022 (Table 1).

**Table 1:** The hyper-parameters for each LGMB regressors for unfiltered and high-passed data.

Hyperparameter	Unfiltered data	High-pass filtered data
Boosting type	gbdt	dart
Colsample bytree	0.672	1
Learning rate	0.020	0.006
Max bin	1000	929
Max depth	256	94
Min child samples	1	17
Min child weight	6.346	4.200
Number of estimators	2697	3539
Number of leaves	179	203
Reg. alpha	0.424	9.8321e-07
Reg. lambda	4.392	1e-09
Subsample	1	0.800
Subsample freq.	8	1
$r^{2}$ (train)	0.999	0.804
$r^{2}$ (test)	0.999	0.697
MSE (train)	0.006	0.492
MSE (test)	0.012	0.670

Following the hyper-parameter optimization for each LightGBM regressor for unfiltered and HP data, we calculated the relative importances based on Permutation Importance algorithm^41^. PI initially calculates a score based on a chosen scoring metric, which in our LGMB regressors chosen as the negative mean squared error, and the best fit LightGBM regressor. This initial score is to be used as a standard measure criterion for the next iterations. Following this step, an independent variable is taken out from the fit, and the scoring metric is recalculated. The difference between the standard measure criterion and the permuted metric without one specific variable is called the PI score of that variable^41^. We repeated the PI calculations for each variable for 100 times to calculate the mean and standard deviation of each variable’s PI based on the best fitting hyper-parameters for each timescale. The same process was also repeated for the negative and positive solar polarity intervals, individually, using the same hyper-parameters found for the unfiltered and high-passed data.

## Data Availability

The datasets used and/or analysed during the current study available from the corresponding author on reasonable request.
